# Monitoring metabolic responses to chemotherapy in single cells and tumors using nanostructure-initiator mass spectrometry (NIMS) imaging

**DOI:** 10.1186/2049-3002-1-4

**Published:** 2013-01-23

**Authors:** Peter J O’Brien, Michelle Lee, Mary E Spilker, Cathy C Zhang, Zhengming Yan, Timothy C Nichols, Wenlin Li, Caroline H Johnson, Gary J Patti, Gary Siuzdak

**Affiliations:** 1 Pfizer Worldwide Research and Development, La Jolla Laboratories, La Jolla, CA, USA; 2 The Scripps Center for Metabolomics and Mass Consortium Corporation, La Jolla, CA, USA; 3 Departments of Chemistry, Genetics, and Medicine, Washington University School of Medicine, St. Louis, MO, USA

**Keywords:** Mass spectrometry imaging, Pharmacodynamic response, Biomarker, Thymidine kinase activity, TK1, 3^′^-Deoxy-3^′^-Fluorothymidine, Tumor proliferation, Nanostructure-initiator mass spectrometry

## Abstract

**Background:**

Tissue imaging of treatment-induced metabolic changes is useful for optimizing cancer therapies, but commonly used methods require trade-offs between assay sensitivity and spatial resolution. Nanostructure-Initiator Mass Spectrometry imaging (NIMS) permits quantitative co-localization of drugs and treatment response biomarkers in cells and tissues with relatively high resolution. The present feasibility studies use NIMS to monitor phosphorylation of 3^′^-deoxy-3^′^-fluorothymidine (FLT) to FLT-MP in lymphoma cells and solid tumors as an indicator of drug exposure and pharmacodynamic responses.

**Methods:**

NIMS analytical sensitivity and spatial resolution were examined in cultured Burkitt’s lymphoma cells treated briefly with Rapamycin or FLT. Sample aliquots were dispersed on NIMS surfaces for single cell imaging and metabolic profiling, or extracted in parallel for LC-MS/MS analysis. Docetaxel-induced changes in FLT metabolism were also monitored in tissues and tissue extracts from mice bearing drug-sensitive tumor xenografts. To correct for variations in FLT disposition, the ratio of FLT-MP to FLT was used as a measure of TK1 thymidine kinase activity in NIMS images. TK1 and tumor-specific luciferase were measured in adjacent tissue sections using immuno-fluorescence microscopy.

**Results:**

NIMS and LC-MS/MS yielded consistent results. FLT, FLT-MP, and Rapamycin were readily detected at the single cell level using NIMS. Rapid changes in endogenous metabolism were detected in drug-treated cells, and rapid accumulation of FLT-MP was seen in most, but not all imaged cells. FLT-MP accumulation in xenograft tumors was shown to be sensitive to Docetaxel treatment, and TK1 immunoreactivity co-localized with tumor-specific antigens in xenograft tumors, supporting a role for xenograft-derived TK1 activity in tumor FLT metabolism.

**Conclusions:**

NIMS is suitable for monitoring drug exposure and metabolite biotransformation with essentially single cell resolution, and provides new spatial and functional dimensions to studies of cancer metabolism without the need for radiotracers or tissue extraction. These findings should prove useful for *in vitro* and pre-clinical studies of cancer metabolism, and aid the optimization of metabolism-based cancer therapies and diagnostics.

## Background

Genetic and environmental cues, including stresses from anti-cancer treatments, can induce significant changes in cell and tissue metabolism. Understanding the relationships between drug exposure and tissue metabolism can improve diagnosis and treatment outcomes, and speed the identification of new drug targets and biomarkers [1]. Quantification of coincident biotransformation of xenobiotics and endogenous metabolites in tumor tissues is critical for understanding exposure-response relationships, but currently demands an impractical degree of analytical sensitivity and spatial resolution. Liquid chromatography-tandem mass spectrometry (LC-MS/MS) characterization of endogenous and xenobiotic metabolites is a cornerstone of drug development, but most methods involve sample extractions that sacrifice spatial resolution for analytical sensitivity. Nanostructure-initiator mass spectrometry imaging (NIMS) is an extension of LC-MS/MS methods that provides mass spectral as well as spatial information from tissue samples.

Thymidine kinase (TK1) activity is an effective and well-established model for monitoring cancer cell cycle status and proliferation potential. This model is ideal for testing the robustness of LC-MS/MS and NIMS analysis for these types of studies as selective metabolite precursors can be assessed, an expansion from early successes using radiotracers to monitor specific metabolites [2]. TK1 activity is tightly linked to both proliferation status and the tumor avidity of thymidine analog tracers [3-8]. It is expressed almost exclusively in the G_1_-S phase of the cell cycle and is significantly elevated in proliferating cells compared to resting or dying cells. TK1 activity can be monitored via cellular retention of phosphorylated thymidine or TK1-selective analogs such as 5-bromo-2^′^-deoxyuridine (BrdU) and 3^′^-deoxy-3^′^-fluorothymidine (FLT) [5]. FLT is taken up by cells and phosphorylated to 3^′^-deoxy-3^′^-fluorothymidine monophosphate (FLT-MP) by TK1. FLT is readily transported out of cells, but FLT-MP is highly retained, and its accumulation can serve as an indirect indicator of proliferating tumor mass. Importantly, TK1 activity and FLT retention are dramatically reduced after efficacious treatment with anti-proliferative drugs [9,10]. Recent reports suggest that mass spectrometry quantification of FLT metabolism to FLT-MP is useful for monitoring the disposition of tumor imaging agents in studies of cellular proliferation without the need for radioactivity, which is required for positron emission tomography (PET) studies with ^18^F]-FLT [11,12].

The potential utility of LC-MS/MS and NIMS as analytical tools in these types of experiments has been indicated by the recent advancements in mass-based metabolite profiling. These advancements have allowed for the analysis of relatively small samples without the need for radiotracers, permitting untargeted analyses of tumor drug responses [1]. In a recent example, the immunosuppressant drug rapamycin was shown to rapidly induce pronounced changes in endogenous metabolism in lymphoid cells by LC-MS/MS [13]. Still, sample preparation for such methods requires tissue extraction, sacrificing anatomical resolution for analytical sensitivity [14,15], highlighting the need for improved metabolomics methodologies. Advancements in mass spectrometry imaging (MSI) and profiling provide promising new tools for metabolomics studies. Some methods are label-free and generate accurate mass measurements across a broad range of analytes. This allows for information-rich, high-specificity biochemical analyses of tissues [16], cells [17,18], and enzyme activity [19].

NIMS is one such improved metabolomics methodology and is a desorption/ionization MSI method that can be used for the analysis of metabolites in single cells and tissues without the need for matrix [20,21]. Thus, sample preparation for NIMS imaging is straightforward, rapid, preserves tissue integrity, and maintains metabolite spatial distribution during image acquisition. These features permit the characterization of dynamic cell and tissue metabolic responses to pharmacological interventions [22]. NIMS thus allows higher resolution quantification of analytes than radiometric imaging and micro-dissection/extraction methods, adding a new dimension for tracking both substrates and metabolic products [19-21]. NIMS can also be used to measure metabolites in single cells [15], raising the possibility that this method might be useful for characterizing tumor drug responses with high resolution.

To explore the utility of NIMS technology for exposure-response studies in cancer drug development, changes in endogenous and xenobiotic metabolism were monitored at the single cell level and in tumor tissues using the TK1 activity model as a proof of principle. LC-MS/MS and NIMS methods were also compared to examine lymphoma and solid tumor anti-proliferative chemotherapy. The results presented here demonstrate that NIMS provides sufficient analytical sensitivity and spatial resolution to detect relevant pharmacodynamic responses in pre-clinical models, and demonstrate the potential of mass-based approaches for optimizing cancer diagnostics and tumor imaging.

## Materials and methods

### Cell culture and drug treatments

Raji Burkitt’s lymphoma cells (CCL-86; ATCC, Manassas, VA, USA) were propagated as per supplier recommendations, in complete growth medium consisting of RPMI-1640 medium supplemented with 10% heat-inactivated fetal bovine serum (FBS, Sigma, St. Louis, MO, USA), 100 IU/mL penicillin, 100 μg/ml streptomycin, and 2mM L-Glutamine (Invitrogen, Carlsbad, CA, USA) at 37°C in a 5% CO_2_ incubator. Cell cultures were maintained at a density of no more than 350,000 cells/mL, and cultured for no more than 30 total passages. Prior to treatment, cells were washed free of complete growth medium and re-suspended in minimum essential medium (MEM) (that is, without FBS) to a density of approximately 100,000 cells/mL. Drug-treated cell cultures were split into aliquots for extraction and LC-MS/MS analysis, or for NIMS analysis as described below. For studies of endogenous metabolite responses to chemotherapy, Raji cells were treated with 50 μM rapamycin or 0.5% (v/v) dimethyl sulfoxide (DMSO) as a vehicle control for up to 90 minutes at 37°C in a 5% CO_2_ incubator. Four experimental replicate samples were prepared. For studies of xenobiotic metabolism, Raji cells were treated with 0.5mM FLT or 0.5% distilled water vehicle control at 37°C in a 5% CO_2_ incubator for 60 minutes. Two experimental replicate samples were prepared, and two technical replicate spots from each of these independent samples were deposited on the NIMS surface.

### LC-MS/MS analysis and metabolite profiling of lymphoma cell extracts

Drug- or vehicle-treated Raji cells were centrifuged at 400 × g for 1 minute and the supernatant was removed. In order to remove confounding media components, cells were washed three times in 1 mL ice cold phosphate-buffered saline (PBS). After the third wash, cells were suspended in 100 μL of extraction solvent containing 10% chloroform, 40% methanol, and 50% nanopure water, and centrifuged at 15,000 × g for 5 minutes. Thus clarified, the supernatant was collected and stored at −80°C for subsequent LC-MS/MS analysis. All Raji cell LC-MS/MS analyses were performed on an Agilent 1200 series high-performance liquid chromatography (HPLC) system coupled to an Agilent 6538 Q-TOF mass spectrometer (Agilent Technologies, Santa Clara, CA, USA) operated in positive electrospray ionization mode. A 4-μL injection volume of rapamycin- or vehicle-treated Raji cell extracts was used. Comparative analysis of rapamycin- and vehicle-treated LC/MS data was performed with XCMS [23] to identify rapamycin-sensitive metabolites, a subset of which were further analyzed by targeted LC-MS/MS using the same HPLC conditions and Q-TOF acquisition parameters. MS/MS spectra were compared to the METLIN metabolite database for metabolite identification [24].

### Single cell NIMS imaging and metabolite profiling

NIMS substrates were prepared as described [25]. In brief, p-type silicon wafers, 500 to 550 μm thick with 0.01 to 0.02 Ω cm resistivity (Silicon Quest International, Santa Clara, CA) were cut into 33 mm^2^ pieces. The wafers were soaked in Piranha solution (sulfuric acid and hydrogen peroxide (2:1)) overnight, washed thoroughly with nanopure water and then dried using nitrogen gas. Etching was carried out by clamping the wafer in a Teflon chamber. Gold foil was used for the anode and a platinum loop as the cathode; a 25% ethanolic hydrogen fluoride solution was then added to the chamber. A BIO-RAD PowerPack1000 (Hercules, CA, USA) was connected and run at a constant-current mode (300 mA) for 30 minutes. The etched wafers were washed in methanol and evaporated to dryness using nitrogen gas. Bis(heptadecafluoro-1,1,2,2-tetrahydrodecyl)tetramethyldisiloxane (Gelest, Morrisvilles, PA, USA) (100 μL) was applied to the surface of the chip and allowed to sit at room temperature for 1 h before using nitrogen gas to remove excess from the surface. The drug- or vehicle-treated Raji cells were washed and centrifuged as described above, and the supernatant was carefully removed. Cell pellets were immediately re-suspended to a density of 5 to 10 cells/μL in cold PBS by gentle pipetting. Thus diluted, cell suspensions were immediately applied to the NIMS surface in 10-μL aliquots and immediately transferred to a desiccated, room temperature vacuum. Sample spots were visibly dry within one minute. NIMS imaging data was acquired at 5-μm intervals using an AB/SCIEX 5800 mass spectrometer in negative-mode. NIMS images of isolated cells were generated using an FLT-MP/FLT intensity ratio, which was calculated for each pixel to generate a ratiometric image. Individual FLT-MP and FLT intensities were calculated as an integrated peak area within a 100-ppm window centered on the calibrated FLT-MP or FLT peak, using Matlab (Version 2010b, The Mathworks Inc., Natick, MA, USA). Single-cell mass spectra and 2-dimensional ratiometric images with four-fold nearest neighbor interpolation were generated with Matlab and 3-dimensional images were generated with Fiji ( http://fiji.sc/). Median normalized total-ion intensity images were generated with Biomap ( http://www.maldi-msi.org/).

### Mouse solid tumor xenograft models, drug treatments, and tissue preparation

All animal experimental procedures complied with the Guide for the Care and Use of Laboratory Animals (Institute for Laboratory Animal Research, 1996) and were approved by the Pfizer Worldwide Research and Development Institutional Animal Care and Use Committee. LC-MS/MS studies of FLT metabolism in tumor tissues were performed using a HCT116 colorectal carcinoma xenograft model. 2.5 million HCT116 cells (CCL247; ATCC) were implanted in the dorsal region of five female Nu/Nu mice (Charles River Breeding Laboratories, Boston, MA, USA) per treatment group. When tumors reached 100 to 120 mm^3^ in volume, typically eight days after implantation, animals received an intra-peritoneal injection of 30 mg/kg docetaxel or vehicle. At 22 or 46 h after docetaxel treatment, each animal received an intra-peritoneal injection of non-radioactive FLT (2μg/kg; approximately 0.2 nanomoles). Two hours later, the animals were euthanized, and tissues were excised then immediately frozen in liquid nitrogen until extraction and LC-MS/MS analysis (see below).

NIMS tissue imaging and immunofluorescence microscopy were performed on tissues excised from SCID-beige mice bearing MDA-MB-231Luc breast tumor xenografts. Two million MDA-MB-231Luc cells (Xenogen Corp., Alameda, CA, USA) were implanted in the dorsal region of female SCID-beige mice (Charles River Labs). When tumors were in the range of 200 to 600 mm^3^, two mice (per group) received an intra-peritoneal injection of 15 mg/kg docetaxel or vehicle 22 hours prior to intra-peritoneal administration of non-radioactive FLT (210 μg/kg; approximately 22 nanomoles).

### LC-MS/MS quantification of FLT and FLT-MP from xenograft tumors and liver tissues

HCT116 xenograft tumors and liver samples were excised and homogenized in methanol at a 3:1 ratio (v/w) using a Mini BeadBeater (BioSPEC Bartlesville, OK, USA). Analytes were extracted from the sample matrix via protein precipitation, through the addition of 250 μL of methanol containing internal standards to 100 μL of reference standard samples, quality control samples, and tissue homogenates [11]. After 15 minutes of centrifugation at 3200 × g and 4°C, the supernatant was transferred to a 1-mL 96-well injection plate, dried under nitrogen at ambient temperature, and reconstituted with 50 μL of injection solvent composed of 0.5mM ethylenediaminetetraacetic acid, and 1 mM citric acid. Chromatographic separation was carried out on a reverse phase analytical column (Phenomenex AQ C18, 100 × 2 mm, 5 μm) coupled with Shimadzu LC-20AC pumps and a SIL-20AC auto-sampler. The mobile phase consisted of solvent A (0.1% formic acid) and solvent B (acetonitrile/0.1% formic acid) at a flow rate of 0.2 mL/minute. A linear gradient of 5 to 30% solvent B was applied during the first 3 minutes. Within 1 minute, solvent B was increased to 70% and held for an additional 0.5 minutes. At the end of the run, solvent B was decreased to 5% in 0.2 minutes, and held at 5% for 2.3 minutes for column re-equilibration. Analytes were then detected on a triple quadrupole mass spectrometer (API4000, Applied Biosystems, Foster City, CA, USA) by monitoring their specific precursor and respective product ions in positive electrospray ionization mode. The mass transitions for FLT, FLT-MP and their correspondent internal standard d_3_-FLT and d_3_-(FLT-MP) were 245.1 → 127.1, 325.3 → 81.2, 248 → 130.2, and 328.2 → 81.2 respectively. For HCT-116 xenograft tumor extracts, FLT-MP peak areas and FLT mass (ng/g tissue) were quantified using d_3_-FLT as an internal standard.

### NIMS xenograft tumor activity imaging

Two hours after FLT administration, MDA-MB-231Luc tumor-bearing animals were sacrificed for tumor harvest, and tumors were frozen in optimum cutting temperature medium (OCT; Sakura Finetek, Torrance, CA, USA) for cryo-sectioning. Frozen OCT-embedded tumors were sliced into 4-μm sections with a cryostat (Leica Microsystems, Buffalo Grove, IL, USA), placed on the NIMS substrate, and immediately dried in a desiccated, room-temperature vacuum chamber. Adjacent tumor sections were collected and placed on glass tissue fixation slides for immunofluorescence microscopy and H&E staining. NIMS imaging data was acquired at 50-μm spatial resolution with an AB/SCIEX 5800 mass spectrometer (Applied Biosystems) in negative-mode across the entire tumor area. Tumor sections from docetaxel-treated and control mice were placed on a single NIMS substrate. A second NIMS chip was used to image tumor sections from the second set of docetaxel-treated and control mice. From the NIMS imaging data, an FLT-MP/FLT ratiometric image was calculated in an identical manner as was done for FLT treated single-cell analysis. In addition, median normalized total-ion intensity, FLT extracted-ion intensity, and FLT-MP extracted-ion intensity images with 4-fold nearest neighbor interpolation were generated in Matlab.

### Optical imaging of xenograft tumors

Double indirect immunofluorescent staining was performed on OCT-embedded, cryostat sections of MDA-MB-231Luc tumor samples. Sections were air-dried, then baked at 37°C for 5 minutes. Sections were fixed in 10% neutral buffered formalin for 10 minutes. Following fixation, sections were washed in 10X Bonds Wash Solution (Leica Microsystems, AR9590) for 5 minutes then blocked in 10% Normal Donkey Serum (#017-000-121, Jackson Immunoresearch, West Grove, PA, USA) for 30 minutes at room temperature. The primary antibodies; anti-Luciferase (#G745A, Promega, Madison, WI, USA, dilution 1:200) and anti-TK1 (ab76495, Abcam, Cambridge, MA, USA, dilution 1:100), were applied to the tissue sections and incubated overnight at 4°C. Sections were washed in Bonds Wash Solution followed by the application of the fluorescent conjugate secondary antibodies applied for 1 h: Alexa Fluor 594 donkey anti rabbit (#A21207, Invitrogen, Carlsbad, CA, USA) and Alexa Fluor 488 donkey anti goat (Invitrogen, #A11055). DAPI (4^′^6- Diamidino-2-phenylindole dihydrochloride, Sigma #32670-5MG-F) was applied for 5 minutes at a dilution of 1:5000 from a working concentration of 5mg/mL as a nuclear counter stain. Slides were overlaid with DAKO Fluorescent mounting medium (S3023, Dako, Carpinteria, CA, USA) for imaging. All immunofluorescent images were generated using the Vectra™, a multispectral microscope slide analysis system (Perkin-Elmer, Waltham, MA, USA), equipped with epi-illumination, a Nuance™ camera, and appropriate filters. Filter cubes used included: (1) UV excitation; 350 nm+/− 25 nm 420 nm long pass emission; (2) blue excitation; 470 nm +/− 20 nm 515 nm long pass emission; (3) green excitation; 535 nm +/− 20 nm 610 nm long pass emission.

## Results and discussion

The present studies were pursued to test the suitability of NIMS for imaging pharmacodynamic changes in tumor metabolism. To demonstrate the selectivity and spatial resolution of the method, NIMS was used to quantify changes in specific endogenous and xenobiotic metabolites single lymphoma cells, first by examining FLT metabolism to FLT-MP, then by monitoring cellular responses to rapamycin. Subsequent studies examined FLT metabolism in solid tumors. In cells and tumors, results obtained using NIMS were comparable with those from LC-MS/MS analysis of cell and tissue extracts. In solid tumors, FLT-MP distribution co-localized with TK1 and tumor-specific antigens, providing additional evidence that cancer cells are the predominant site of FLT metabolism in these tumors. The utility of NIMS for simultaneously characterizing exposure to drugs and tracers should facilitate the optimization of *in vitro* proliferation assays and (^18^F)-FLT PET tumor imaging, which in turn should aid the identification of complementary measures of tumor drug responses.

### Mass spectrometry imaging of metabolism in single cells

TK1-mediated metabolism was chosen as a model system for monitoring drug exposure and pharmacodynamic responses. Some of the most commonly used cell proliferation assays measure cellular retention of thymidine or TK1-selective analogs, such as (^3^H)-Thymidine, BrdU, and (^18^F)-FLT. The cellular retention of these entities correlates with intracellular TK1 expression [26]. TK1 is expressed almost exclusively in G_1_-S phase cells, and treatment-induced changes in tracer retention are often interpreted as alterations in cell cycle progression or cell viability. However, most of these assays do not account for the behavior of the parent tracer, which varies across cell lines and tissues due to cell culture conditions, the actions of transporter inhibitors, and off-target drug effects [27,28]. Our previous work using LC-MS/MS clearly demonstrates that mass spectrometry can quantitatively detect the conversion of tracer amounts of FLT to FLT-MP [11]. We therefore used NIMS to measure FLT metabolism in single cells.

Raji Burkitt’s lymphoma cells are highly proliferative, and thus express high endogenous levels of TK1 [29,30]. Here, Raji cells were treated with 0.5 mM FLT or vehicle for 60 minutes, after which FLT metabolism to FLT-MP was analyzed using NIMS. This dose of FLT is well above tracer concentrations and was selected to assure sufficient conversion of FLT to FLT-MP for detection of single cells using NIMS. FLT is an extremely potent anti-retroviral drug that is relatively well tolerated by cells and animals exposed to high doses over short periods of time [31,32]. Since FLT competes with endogenous thymidine, it is expected to have direct effects on thymidine metabolism. To demonstrate NIMS suitability for monitoring more distal pharmacodynamic responses, Raji cell responses to the starvation mimetic immunosuppressant drug, rapamycin, were also monitored using NIMS. In both cases cells were pelleted by centrifugation, re-suspended in PBS to a density of approximately 5 to 10 cells/μL, then immediately applied to a NIMS surface and dried under vacuum. This dilution procedure reduces cellular aggregation on the NIMS surface (our unpublished observations). The rapid drying process and analysis under high vacuum helps to arrest post-collection cellular metabolism, and the fluorinated surface of NIMS chips limits the dispersal of metabolites sufficiently to distinguish single cells from matrix contaminants [15]. In this study, due to the small cellular diameter and relatively low number of cells on the NIMS surface, NIMS spectra were generated using a 5-micron step size, which also provides higher resolution of cellular geometry.

**Figure 1:**
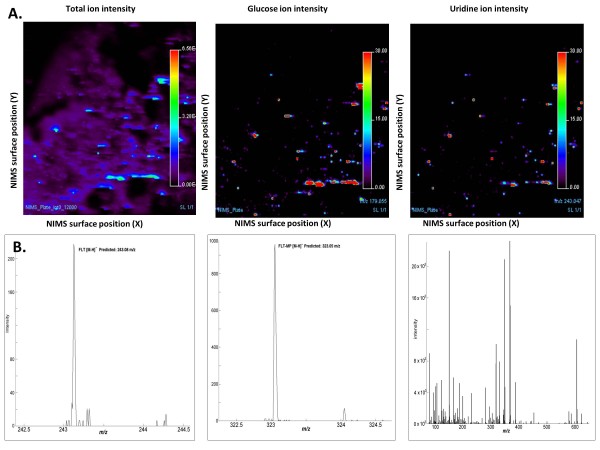
**Single cell endogenous and xenobiotic metabolite detection using nanostructure-initiator mass spectrometry (NIMS). **NIMS total ion intensity (panel **A**, left), glucose ion intensity (center) and uridine ion intensity (right**) **of 3^′^-deoxy-3^′^-fluorothymidine (FLT)-treated Raji cells. The total-ion NIMS image indicates that numerous cells are present in the same regions on the NIMS surface as suggested by high-intensity localized signals. **(B) **Typical NIMS mass spectra observed from a pixel corresponding to a putative single cell in the NIMS Raji cell images. Left panel, FLT peak [M-H] 243.08 m/z) from a single cell; center, FLT-MP [M-H]323.05 m/z) peak from a single cell; right, single-pixel NIMS spectrum across the 75 to 650 m/z range.

Mass spectrometry images of FLT-treated Raji cells show numerous 10- to 15-micron regions with elevated ion intensity (Figure 1A, left panel). The size and spectral complexity of these features is consistent with the presence of Raji cells on the chip surface. The total ion intensity in these putative cells after treatment with FLT, vehicle or 50 μM rapamycin can also be observed in Figure S1 A, B, and C respectively, in Additional file 1. In order to further distinguish individual cells amongst higher intensity non-specific ions, NIMS images were also generated for glucose and uridine. The signal intensities for glucose (Figure 1A, middle panel) and uridine (right panel), respectively, are in the same positions on the 2-dimensional NIMS surface, outlining putative cells. Figure S1 in Additional file 1 also shows that the concentrations of these endogenous metabolites did not change after FLT or rapamycin treatment. Careful examination of these figures further shows that the most intense spots in the total ion intensity images do not necessarily overlap with uridine and glucose single ion intensity images, highlighting the value of single ion imaging for discriminating specific metabolic features in individual cells and tissues. This co-localization of high concentrations of water-soluble endogenous metabolites with regions of high spectral complexity supports the conclusion that these spectra arise from single cells. These figures also show that the cellular intensities of metabolically distinct ions can vary enough that differences in signal threshold settings can lead to loss of signal from some cells. In FLT-treated samples, however, the presence of FLT-MP reflects both thymidine kinase activity and exposure to FLT. Mass spectra taken from an individual pixel show both FLT (Figure 1B, left panel) and FLT-MP (center panel) as well as the m/z range 75 to 650 (right panel). The breadth and complexity of these mass spectra demonstrate the exquisite specificity of mass-based analysis using NIMS and highlight the potential value of simultaneous measurement of parent drugs and their metabolites for identifying tissue structural features with cell-level resolution.

**Figure 2:**
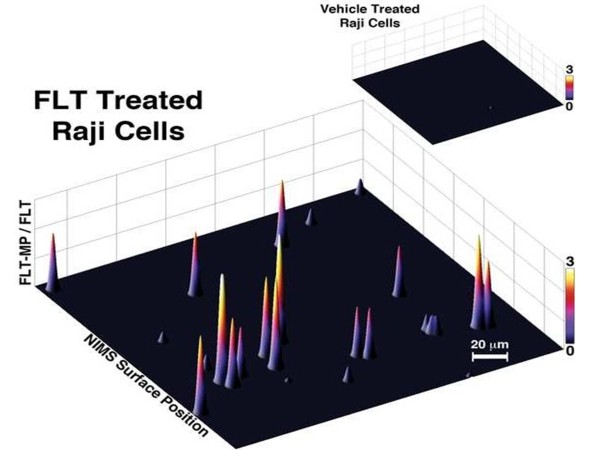
**Nanostructure-initiator mass spectrometry (NIMS) single-cell FLT-MP/FLT ratiometric imaging.** Ratiometric analysis indicates no FLT-MP/FLT intensity in untreated Raji Cells (top right), and significant FLT-MP metabolite production and retention in FLT-treated Raji Cells (bottom). Cells were plated sparsely to ensure that single cells were imaged. FLT, 3^′^-deoxy-3^′^-fluorothymidine; FLT-MP, 3^′^-Deoxy-3^′^-Fluorothymidine monophosphate.

**Figure 3:**
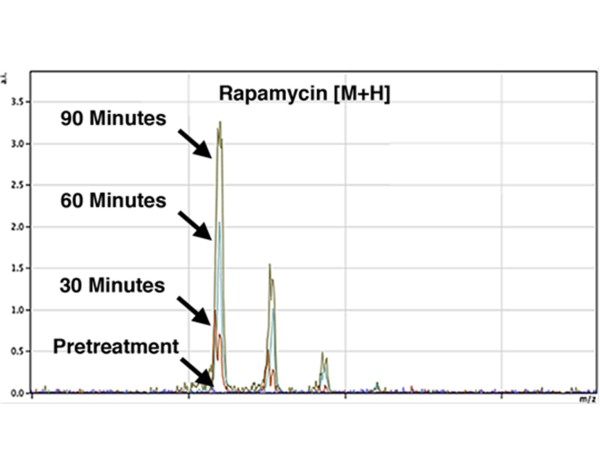
**Nanostructure-initiator mass spectrometry (NIMS) detection of rapamycin pharmacokinetics in Raji cells. **Ninety-minute treatment shows increased drug uptake over time.

Although FLT cellular retention is primarily driven by TK1 activity, TK1 substrate availability and the catabolism of its reaction end products can affect FLT accumulation [12,27,28,33,34]. We sought to correct for these factors and potential spectrum-wide intensity variation that can result from localized inhomogeneity within the sample on the NIMS surface. Specifically, we monitored both FLT and FLT-MP in order to generate ratiometric NIMS images of TK1 activity for each pixel. In Figure 2, regions with high TK1 activity (that is, regions with high FLT-MP/FLT ratios) in the FLT-treated sample are associated with cells, a distribution that likely reflects intracellular FLT biotransformation mediated by TK1. The individual peaks shown in Figure 2 thus represent the 3-dimensional FLT-normalized FLT-MP peak intensities from single cells. Most cells showed considerable accumulation of FLT-MP relative to FLT. This rapid conversion of FLT to FLT-MP reflects significant competition with endogenous thymidine and suggests that the high dose of FLT used in these NIMS studies might have pharmacological effects. Indeed, examination of NIMS and LC-MS/MS metabolite profiles revealed significant dysregulations in thymidine metabolism after only brief high dose FLT treatments (data not shown). Therefore, in order to demonstrate the suitability of NIMS for measuring downstream pharmacodynamic responses, Raji cells were treated with rapamycin, a potent immunosuppressant and starvation mimetic that causes rapid dysregulations in cellular metabolism [13]. Raji cell cultures were exposed to 50 μM rapamycin or vehicle for up to 90 minutes and then split into aliquots for analysis by NIMS single-cell imaging or LC-MS/MS. Rapamycin was readily detectable by NIMS, and cellular concentrations of rapamycin increased across the entire 90-minute time course (Figure 3). Consistent with the results of Ramanathan and Schreiber [13], NIMS metabolite profiling of 60-minute samples showed significant dysregulation of an array of endogenous metabolites, including several with identifiable MS/MS fragmentation patterns that were confirmed using LC-MS/MS (Table 1). This finding demonstrates that NIMS has adequate sensitivity to detect both drugs and endogenous biomarkers at the single cell level. Taken together, these results confirm that NIMS provides a highly accurate method for measuring the cellular conversion of specific metabolites, providing a new dimension for analyzing cell metabolism and tumor drug responses.

**Table 1:** Selected rapamycin-sensitive metabolites identified by nanostructure-initiator mass spectrometry (NIMS)

**Metabolite**	**Metabolite median fold change**	***P*-value**
Tigylglycine	3.4	0.0112
N-acetyl-glutamic acid	10	0.0331
Uridine monophosphate	2.0	0.0021
Isobutyrylcarnitine	2.3	0.0368
Stearic acid	4.7	0.0893
D-glucose	2.3	0.0713

Raji cells were treated with Rapamycin or vehicle for 60 minutes as described in Materials and Methods, then divided into matching aliquots for NIMS analysis. Metabolites with unambiguous MS/MS fragmentation patterns that represent disparate metabolic pathways were also quantified and compared to reference standards via LC-MS/MS, and are shown here.

### LC-MS/MS quantification of solid tumor FLT metabolism

**Figure 4:**
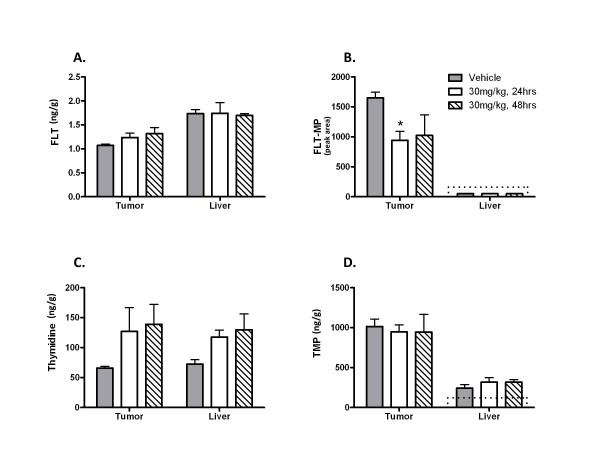
**Liquid chromatography-tandem mass spectrometry (LC-MS/MS) analysis of 3**^**′**^**-deoxy-3**^**′**^**-fluorothymidine (FLT) and 3**^**′**^**-deoxy-3**^**′**^**-fluorothymidine monophosphate (FLT-MP) accumulation in xenograft tumors and liver extracts from HCT116 tumor-bearing mice. **FLT is distributed equally well in tumors and non-proliferative liver control tissues from all treatment groups (ng FLT/g tissue, panel **A**). FLT-MP is detectable in tumor tissues, but not in liver controls (panel **B**, FLT-MP peak area). Compared to vehicle-treated tumors (gray bars, n = 5) FLT-MP is significantly reduced 24 h (white bars; n = 4; *P *= 0.009, Student’s *t-*test) and 48 h (hashed bars, n = 4; *P *= 0.165) after docetaxel treatment. FLT-MP concentrations were below the validated lower limit of quantitation of the assay in matched liver extracts (dotted lines) from all treatment groups. Thymidine and thymidine monophosphate (TMP) were detectable in all tissues (panels **C **and **D**). Results represent means +/− SD for each treatment group.

Tumor imaging using (^18^F)-FLT PET is an established method for linking tumor responses to anatomy, and provides a powerful tool for drug discovery [5]. However, this method requires a radioactive label for detection, and cannot discriminate between the parent tracer and its metabolites. Mass spectrometry quantification of FLT metabolism to FLT-MP by NIMS eliminates the need for radioactivity and can also provide useful information on the spatial localization and disposition of tracers, drugs, and other metabolites. To demonstrate that mass spectrometric monitoring of TK1 activity is suitable for studying pharmacodynamic responses in xenograft tumors, nude mice implanted with HCT116 colorectal carcinoma cells were treated with 30 mg/kg docetaxel for 22 or 46 h, then given a tracer dose of non-radioactive FLT. After 2 h, the animals were sacrificed and tumors were excised along with livers, which served as a non-proliferative tissue control. Tumor extract FLT and FLT-MP were analyzed by LC-MS/MS using the methods of Li *et al.*[11]. As shown in Figure 4A, FLT distributed equally well into tumor and liver tissues regardless of treatment group. FLT-MP accumulation was also readily detected in tumor tissues (panel B), but FLT-MP was below the assay limit of detection in liver. Furthermore, FLT-MP accumulation was significantly higher in tumors from vehicle-treated animals when compared to samples collected 24 and 48 hours after docetaxel treatment; a result that is consistent with the well characterized anti-proliferative effects of this drug [35]. Thymidine and its phosphorylated metabolite thymidine monophosphate (TMP) were detectable in both tumors and liver, likely reflecting the activity of TK2, the predominant thymidine kinase in quiescent and dying cells [36]. Taken together, these data show that mass spectrometry is suitable for measuring changes in endogenous and xenobiotic metabolism (that is, the conversion of thymidine to TMP and FLT to FLT-MP) as an indicator of solid tumor drug responses. While these studies were underway, Zhang and colleagues described a comparison of (^18^F)-FLT PET imaging of xenograft tumors with LC-MS/MS measurement of FLT metabolism [12]. That study demonstrated a direct correlation between (^18^F)-FLT tracer avidity and tumor accumulation of non-radioactive FLT-MP, and revealed that MDA-MB-231Luc breast tumor xenografts had more favorable growth characteristics and FLT tracer avidity than HCT116 xenografts. Those findings led us to use the MDA-MB-231Luc model for the NIMS studies described below.

### NIMS quantification of solid tumor FLT metabolism

**Figure 5:**
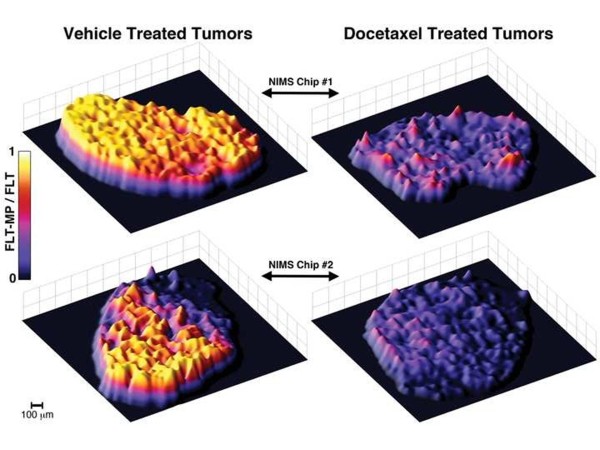
**Nanostructure-initiator mass spectrometry (NIMS) FLT-MP/FLT ratiometric imaging of docetaxel- and vehicle-treated breast tumor mouse xenografts. **Three-dimensional NIMS images of tumor sections from 3^′^-deoxy-3^′^-fluorothymidine (FLT)-treated mice shows significantly higher conversion of FLT to FLT-MP in vehicle-treated (left) versus docetaxel-treated mice (right). Tumor sections from one set of vehicle- and docetaxel-treated mice were placed in close proximity on a single NIMS chip and imaged using NIMS (Chip #1, top). As further validation, tumor sections from a second set of vehicle- and docetaxel-treated mice were placed in close proximity on a second NIMS chip and imaged using NIMS (Chip #2, bottom). A total of four independent tumors were imaged, two from docetaxel-treated and two from vehicle-treated mice. FLT-MP/FLT ratios were normalized to a maximum value of 1 for each NIMS chip.

**Figure 6:**
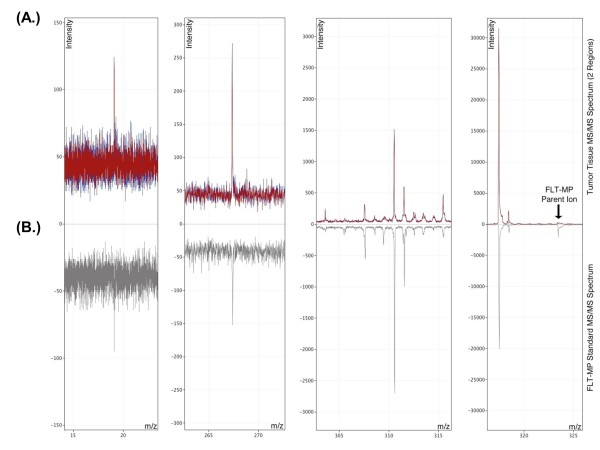
**Comparison of the nanostructure-initiator mass spectrometry (NIMS) tandem mass spectrometry (MS/MS) fragmentation pattern of a 3**^**′**^**-deoxy-3**^**′**^**-fluorothymidine-monophosphate (FLT-MP) reference standard with that of putative FLT-MP from tumor tissues.** The FLT-MP fragmentation pattern [M-H] 323.05 m/z) acquired *in situ *from two separate tumor sections (panel **A, **two spectra overlay) matches that of an FLT-MP reference standard spotted adjacent to the tumor edge on the same NIMS chip (panel **B**). The reference standard fragmentation spectrum is displayed inverted to enable a more direct comparison of tumor-derived spectra with that of the reference standard.

MDA-MB-231Luc tumor-bearing mice were treated with vehicle or an efficacious dose of docetaxel for 22 h, and then given an intra-peritoneal injection of non-radioactive FLT. The dose used in these proof-of-principle studies is approximately 100 times that used in the HCT-116 experiments described above, and was chosen to assure that FLT would be detectable in at least some tumor regions without undue toxicity. Two hours after FLT treatment, tumors were harvested, embedded in OCT medium and frozen. Four-micron sections were cut from the harvested tumors, placed on the NIMS surface, and immediately dried under vacuum. NIMS imaging of tumor sections was performed with 50 μm spatial resolution and FLT-MP/FLT ratiometric images were generated (Figure 5 and Additional file 2). This step size was chosen to facilitate speed of analysis. Differences in total ion scans indicate metabolic inhomogeneity within each tissue slice, but these differences are confined to specific areas and separated by distances on a scale of several hundred microns, which is resolvable by the 50-μm step size used for NIMS imaging. Variations in the FLT-MP/FLT ratio across the tissue were likely minimized because ratiometric images suggest general metabolic homogeneity across the tissue. Consistent with docetaxel pharmacology, total ion intensity NIMS images and FLT-MP ratiometric NIMS images from vehicle- (Additional file 2: Figure S2A and C) and docetaxel-treated tumors (Additional file 2: Figure S2B and D) clearly distinguish aggressively growing vehicle-treated tumors from docetaxel-treated tumors. Reduced FLT-MP in docetaxel-treated mice is also apparent from FLT-MP extracted ion intensity images, whereas limited differences are observed in FLT extracted ion and total ion images from vehicle and docetaxel-treated mice. *In situ* MS/MS fragmentation data was also collected to verify NIMS accurate mass measurements against a pure FLT-MP reference standard applied to the same NIMS surface just outside the tumor edge. The NIMS MS/MS spectrum obtained from tumor sections matches the fragmentation pattern observed from pure FLT-MP standard (Figure 6). Taken together, these NIMS tissue imaging results are consistent with the LC-MS/MS results described above for HCT116 xenografts as well as previous reports examining (^18^F)-FLT metabolism the MDA-MB-231Luc model [12]. In all cases, FLT-MP is more abundant in vehicle-treated xenograft tumors compared to docetaxel-treated tumors.

### TK1 and tumor antigen distribution in xenograft tumors

The distribution of TK1 protein and tumor cell line-specific luciferase was examined using immunofluorescence microscopy (IFM) of adjacent tissue sections from vehicle-treated mice (Additional file 3). In these samples, TK1 immunoreactivity can arise from proliferating murine cells or xenograft tissues, while luciferase is expressed solely in xenograft tumor cells. H&E staining showed that homogenously distributed tumor cells make up the majority of vehicle-treated xenograft tumors, and that docetaxel treatment caused widespread necrosis in treated tumors (data not shown). IFM of vehicle-treated tumors shows widespread luciferase immunoreactivity indicative of xenograft cells. Luciferase consistently co-localizes with both nuclear DAPI staining and TK1 immunoreactivity, indicating that most of the tumor mass consists of proliferating, xenografted cells. Despite the absence of a direct comparator, TK1 and tumor cell line-specific luciferase were co-localized in control tumors in a pattern similar to that of the highest FLT-MP/FLT ratios, further implicating tumor-derived TK1 in the metabolism of FLT to FLT-MP.

### Study limitations

In our NIMS lymphocyte experiments, we describe metabolite-rich regions as putative cells, but it is possible that FLT might leak from cells during desiccation on the NIMS surface, leading to artifactual accumulation of metabolites in cell-sized spots. The abundance and consistent co-localization of multiple highly water-soluble analytes across cell populations in different treatment groups argues against this and suggests that if there is leakage of metabolites from the cells, it is remarkably consistent. Furthermore, the cell washing, dilution, and plating protocols described here likely reduce contamination from media components, including metabolites released from cells during sample preparation [15].

The pharmacology of high dose FLT may also confound our Raji cell FLT studies, and may limit the usefulness of these feasibility studies for optimizing (^18^F)-FLT PET methods. Published reports describe the toxicity of azidothymidine (AZT), FLT, and other anti-retroviral drugs in cell lines and mice [31,32]. Those studies show that FLT is generally well-tolerated at lower doses, but that cytotoxicity and bone marrow suppression are seen at high, sustained FLT concentrations, likely due to effects on DNA replication (3-day cytotoxicity IC_50_ = 240 μM) [31]. Given that, we chose to use this relatively high FLT exposure with a shorter incubation time to enhance the likelihood that FLT-MP could be detected in single cells.

Given the observed metabolic dysregulations induced in Raji cells by FLT and rapamycin treatment, it is also important to consider the potential effects of temperature shifts, centrifugation, osmotic and oncotic shifts, and other experimental manipulations on cell biology. Our experimental designs and sample handling protocols for both *in vitro* and *in vivo* studies attempted to minimize the impact of sample handling on xenobiotic and endogenous metabolism, but practical limitations of the method no doubt introduce some degree of uncontrolled variability into our results.

Our *in vivo* studies were designed to test the technical feasibility of NIMS imaging for monitoring pharmacodynamic responses to cancer chemotherapy. Although NIMS clearly shows drug-induced changes in FLT metabolism in docetaxel-treated mice, these studies were not designed to address the selectivity and specificity of these methods for TK1 activity, cell cycle status, nor the mechanisms that drive the observed changes in FLT metabolism. Instead, we relied on published reports and our own experience to design dosing and sampling strategies. Docetaxel elicits reproducible and predictable changes in radiotracer, bioluminescence and immunohistochemical endpoints in MDA-MB-231luc xenograft models [12,37-48]. TK1 expression and treatment-induced changes in TK1 activity, reflected by (^18^F)-FLT tracer, FLT tumor avidity and kinetics, are also quite predictable in the model systems used here. In both MDA-MB-231 and Burkitt’s lymphoma xenografts it was shown that FLT uptake two hours after injection (the same time point used in our *in vivo* studies) varies by less than 25% [47]. Considering these factors, we limited the present feasibility study to two mice per treatment group. The results shown in Additional file 2 and in numerous publications [2-6,8-10,27,29,49-61], suggest that the sample sizes used for these studies are appropriate for the current application.

With regard to the specificity of this approach for measuring TK1 activity, extensive literature describes the cell biology of TK1 and its critical role in the differential disposition of FLT and other thymidine analogs [2-6,8-10,27,29,49-61]. Those reports and others indicate that the drug-induced changes in FLT metabolism reported here likely reflect alterations in intracellular TK1 abundance, but this question was not directly addressed. Manipulation of TK1 expression in isogenic cells would provide an additional test of the linkage between TK1 expression and changes in the FLT-MP/FLT ratio after drug treatment. Two recent publications address this issue in relevant biological models. First, Barthel and colleagues [56] examined (^18^F)-FLT uptake *in vivo* using L5178Y mouse lymphoma cells containing the functional heterozygous TK1^+/−^ allele and a corresponding TK1^−/−^ L5178Y variant derived from mutation of the TK1 locus. The report demonstrated that TK1 protein expression drives (^18^F)-FLT uptake in lymphoma cells. Secondly, Zhang and colleagues addressed the linkage between TK1 expression and FLT tracer uptake in drug-treated mice [12]. Mice bearing MDA-MB-231Luc xenografts were treated with the cell cycle inhibitor drug PD-0332991, which caused cell cycle arrest, a reduction in BrdU incorporation, reduced Ki-67 and TK1 protein expression, and lowered (^18^F)-FLT uptake in tumor tissues. Drug washout led to a return to asynchronous growth, return of proliferation antigens, and an increase in (^18^F)-FLT uptake. Taken together, these reports further support the assertion that the changes in FLT-MP/FLT ratios seen in the present study are due to treatment-induced alterations in TK1 activity. It is important to note, however, that the NIMS approach described here does not discriminate between TK1 loss from dying cells (for example, via enzyme leakage) and other modes of downregulation in cycling, viable cells. Although regions of necrosis were clearly seen in the tumor sections from docetaxel-treated cells, it remains possible that the observed reduction in TK1 activity shown in these studies is not entirely due to cytotoxicity. One long-term goal of these studies is to identify endogenous metabolic markers for discriminating non-proliferative but viable cells from dead and dying cells. Our experiments with cultured Raji cells suggest that NIMS imaging of water-soluble endogenous metabolites like glucose and uridine might help to identify intact, non-proliferating tumor cells. Given the pleiotropic cytotoxicity of docetaxel [62,63], reliable interpretation of such data would require more careful monitoring of tumor drug exposure than was pursued in the present studies.

## Conclusions

The results described here demonstrate that NIMS can be used to detect drug exposure (that is, intracellular uptake of FLT and rapamycin), xenobiotic metabolism (FLT conversion to FLT-MP), and downstream pharmacodynamic responses at the level of single cells. NIMS should prove useful for further characterizing these cellular responses and additional dissection of this biology. Given the single-cell sensitivity of NIMS demonstrated here, ratiometric imaging should enhance investigations of metabolic heterogeneity in cells coincident with other metabolic profiles. This tool would thereby provide a means to identify endogenous metabolites with differential sensitivity to chemotherapy, some of which might serve as novel indicators of cellular proliferation. The identification of endogenous metabolite fingerprints associated with different tissues and cell fates would also eliminate issues associated with non-tracer doses of FLT, like those used in the present studies. Ratiometric measurement of an administered compound and its corresponding metabolite should be generally applicable to MSI studies of xenobiotic biotransformation in animal models, particularly in cases where metabolites are retained to some extent in cells of interest [22]. This would permit MSI signal normalization to endogenous metabolites instead of xenobiotics, which would further improve metabolomic characterization of treatment responses. The single-cell sensitivity demonstrated here should also improve proof-of-concept studies in cell culture prior to more expensive and time-consuming pharmacodynamic studies in animals.

The clinical utility of these methods remains to be seen, since the present studies focused entirely on cultured cells and excised tissues. The present studies demonstrate that NIMS likely has adequate sensitivity and resolution to analyze cells from the circulation and patient tissue biopsies. These methods may therefore have immediate utility in studies of hematological malignancies or studies of circulating tumor cells. Sampling via fine needle biopsy, for example, may allow for serial studies of FLT metabolism, particularly in lymphoma and other liquid tumors. Finally, since alterations in FLT metabolism can reflect cellular changes that dramatically affect the disposition and pharmacological activity of therapeutic nucleoside analogs [64], the methods described here should also aid the optimization of existing anti-cancer therapies.

## Abbreviations

BrdU: 5-bromo-2^′^-deoxyuridine; DAPI: 4^′^,6-diamidino-2-phenylindole; DMSO: Dimethyl sulfoxide; FBS: Fetal bovine serum; FLT: 3^′^-deoxy-3^′^-fluorothymidine; FLT-MP: 3^′^-deoxy-3^′^-fluorothymidine monophosphate; H&E: Haematoxylin-eosin; HPLC: High-performance liquid chromatography; IFM: Immunofluorescence microscopy; LC-MS/MS: Liquid chromatography-tandem mass spectrometry; MEM: Minimum essential medium; MSI: Mass spectrometry imaging; NIMS: Nanostructure-initiator mass spectrometry; OCT: Optimum cutting temperature; PBS: Phosphate-buffered saline; PET: Positron emission tomography; TK1: Thymidine kinase; TMP: Thymidine monophosphate.

## Competing interests

The authors declare no competing interests.

## Authors’ contributions

PJO’B conceived the study and wrote the manuscript; MES, CZ, ML, TCN, GS, and PJO’B designed the experiments. ZY performed the animal experiments, ZY and WL performed the LC-MS/MS experiments on solid tumor extracts. IFM experiments were performed by ML. ML, MES, CZ, TCN, WL, CHJ, GJP, GS, and PJO’B analyzed and/or interpreted the data. All authors commented on the manuscript. All authors read and approved the final manuscript.

## Supplementary Material

Additional file 1 **Figure S1. **Total ion, glucose ion, and uridine ion intensity nanostructure-initiator mass spectrometry (NIMS) images of Raji cells. (A) FLT-treated cells, (B) vehicle treated cells, and (C) rapamycin-treated cells. Equally scaled total ion NIMS images indicate that numerous cells are present in the same regions on the NIMS surface as suggested by high-intensity localized signals. Note that the vehicle-treated NIMS chip has 87% background intensity of the FLT-treated chip. Click here for file

Additional file 2 **Figure S2. **Total ion nanostructure-initiator mass spectrometry (NIMS), 3^′^-deoxy-3^′^-fluorothymidine (FLT) and 3^′^-deoxy-3^′^-fluorothymidine monophosphate (FLT-MP) extracted ion NIMS, and FLT-MP/FLT ratiometric NIMS tumor images. Tumor section images from the four mice used in this study, two vehicle-treated mice and two docetaxel-treated mice. Tumor sections from **(A) **vehicle-treated and **(B)** docetaxel-treated mice imaged on a single NIMS chip. Tumor sections from two additional **(C)** vehicle-treated and **(D) **docetaxel-treated mice on a second NIMS chip. NIMS total-ion and extracted-ion images are normalized and scaled for comparison within a particular column. Columns (from left) show total ion Intensity, FLT [M-H] 243.08 m/z) extracted ion intensity, FLT-MP [M-H] 323.05 m/z) extracted ion intensity, and FLT-MP/FLT ratiometric magnitude. Click here for file

Additional file 3 **Figure S3. **Immunofluorescence images of tumor sections. Images of vehicle-treated samples were generated from 4-μm-thick tumor tissue slices adjacent to the tumor sections used to acquire the nanostructure-initiator mass spectrometry (NIMS) images. Representative images at 20× magnification from vehicle-treated tumors (top panel: NIMS Chip 1 vehicle tumor; bottom panel: NIMS Chip 2 vehicle tumor). Displayed areas were selected to be representative of viable tumor regions based on DAPI staining (blue); in addition TK1 and anti-luciferase immunoreactivity (red and green, respectively) are shown. Click here for file
