# Supplementary material for: Monitoring metabolic responses to chemotherapy in single cells and tumors using nanostructure-initiator mass spectrometry (NIMS) imaging
Source: Cancer Metab. 2013 Jan 23;1:4. doi: 10.1186/2049-3002-1-4 (PMC3834492; doi:10.1186/2049-3002-1-4)
Supplement: Additional file 1 — Figure S1. Total ion, glucose ion, and uridine ion intensity nanostructure-initiator mass spectrometry (NIMS) images of Raji cells. (A) FLT-treated cells, (B) vehicle treated cells, and (C) rapamycin-treated cells. Equally scaled total ion NIMS images indicate that numerous cells are present in the same regions on the NIMS surface as suggested by high-intensity localized signals. Note that the vehicle-treated NIMS chip has 87% background intensity of the FLT-treated chip. [file 2049-3002-1-4-S1.pptx]

## Slide 1
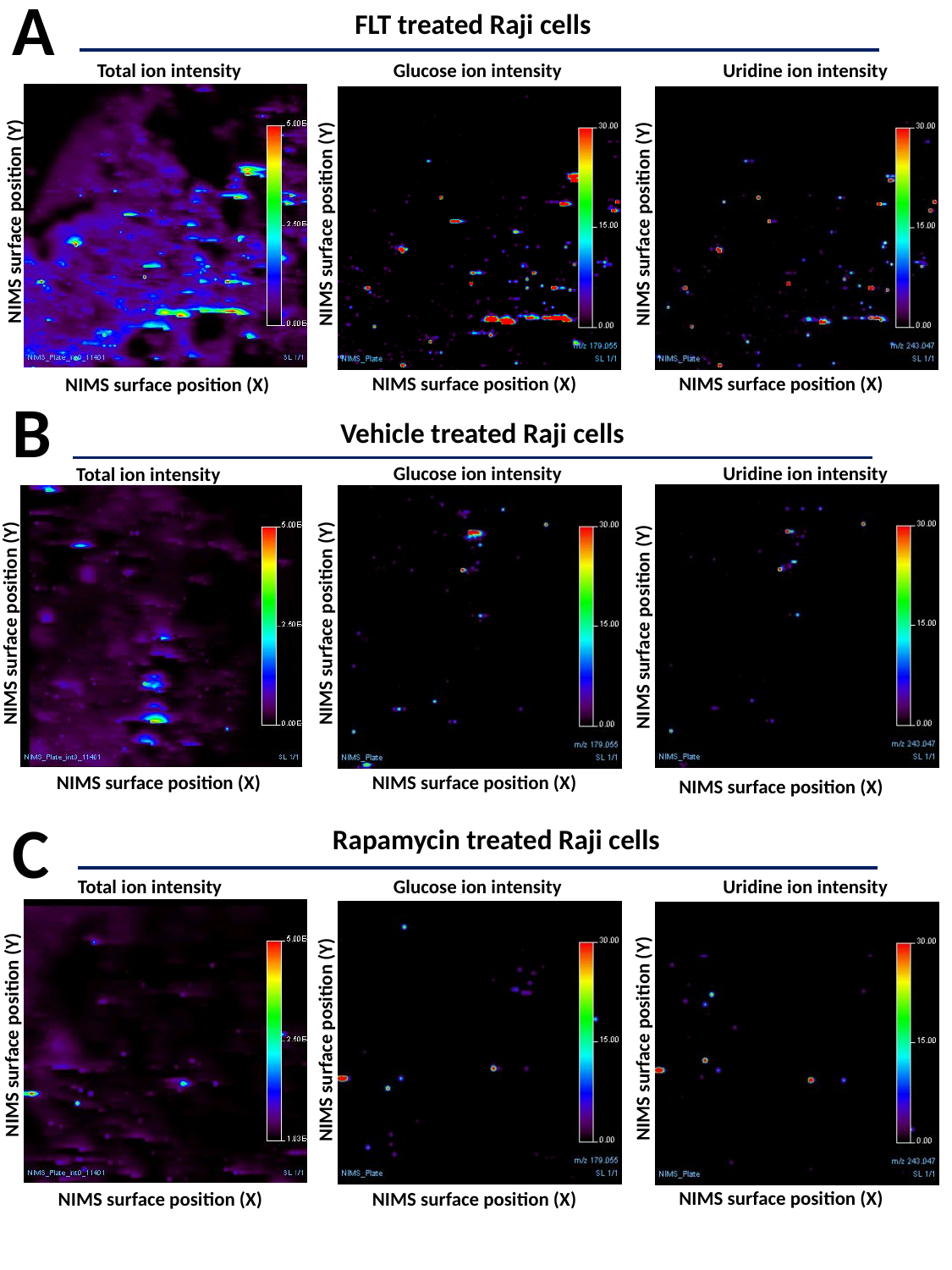

FLT treated Raji cells
A
Total ion intensity
NIMS surface position (Y)
NIMS surface position (X)
Glucose ion intensity
NIMS surface position (Y)
NIMS surface position (X)
Uridine ion intensity
1 CELL
NIMS surface position (Y)
2 CELLS
NIMS surface position (X)
B
Vehicle treated Raji cells
Glucose ion intensity
Uridine ion intensity
Total ion intensity
NIMS surface position (Y)
NIMS surface position (Y)
NIMS surface position (Y)
NIMS surface position (X)
NIMS surface position (X)
NIMS surface position (X)
C
Rapamycin treated Raji cells
Total ion intensity
Glucose ion intensity
Uridine ion intensity
NIMS surface position (Y)
NIMS surface position (Y)
NIMS surface position (Y)
NIMS surface position (X)
NIMS surface position (X)
NIMS surface position (X)
