# Supplementary figures and images for: Monitoring metabolic responses to chemotherapy in single cells and tumors using nanostructure-initiator mass spectrometry (NIMS) imaging
Source: Cancer Metab. 2013 Jan 23;1:4. doi: 10.1186/2049-3002-1-4 (PMC3834492; doi:10.1186/2049-3002-1-4)

## Slide 1
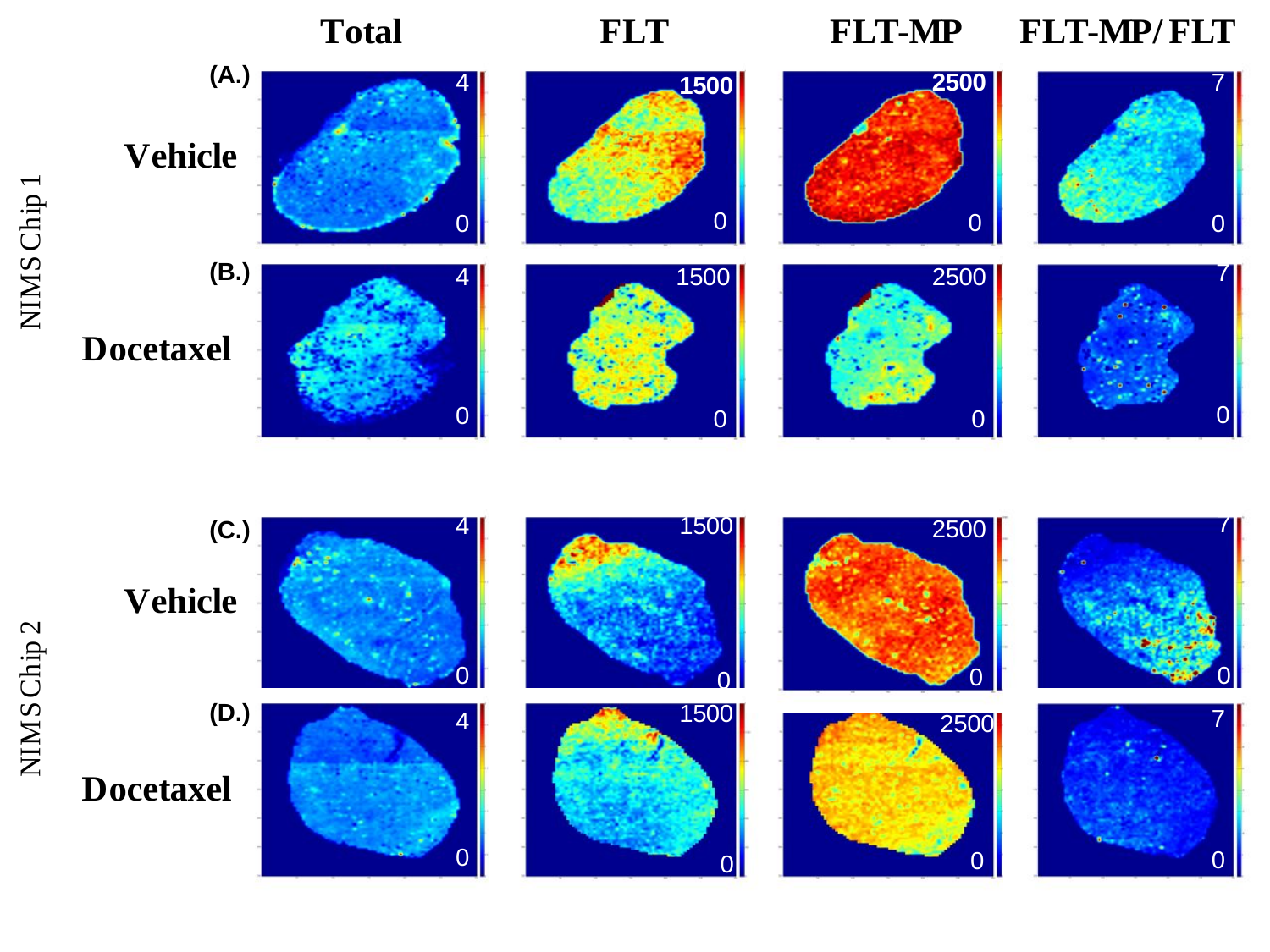

(A.)
(B.)
(C.)
(D.)

Supplement: Additional file 2 — Figure S2. Total ion nanostructure-initiator mass spectrometry (NIMS), 3′-deoxy-3′-fluorothymidine (FLT) and 3′-deoxy-3′-fluorothymidine monophosphate (FLT-MP) extracted ion NIMS, and FLT-MP/FLT ratiometric NIMS tumor images. Tumor section images from the four mice used in this study, two vehicle-treated mice and two docetaxel-treated mice. Tumor sections from (A) vehicle-treated and (B) docetaxel-treated mice imaged on a single NIMS chip. Tumor sections from two additional (C) vehicle-treated and (D) docetaxel-treated mice on a second NIMS chip. NIMS total-ion and extracted-ion images are normalized and scaled for comparison within a particular column. Columns (from left) show total ion Intensity, FLT [M-H] 243.08 m/z) extracted ion intensity, FLT-MP [M-H] 323.05 m/z) extracted ion intensity, and FLT-MP/FLT ratiometric magnitude. [file 2049-3002-1-4-S2.pptx]

## Slide 1
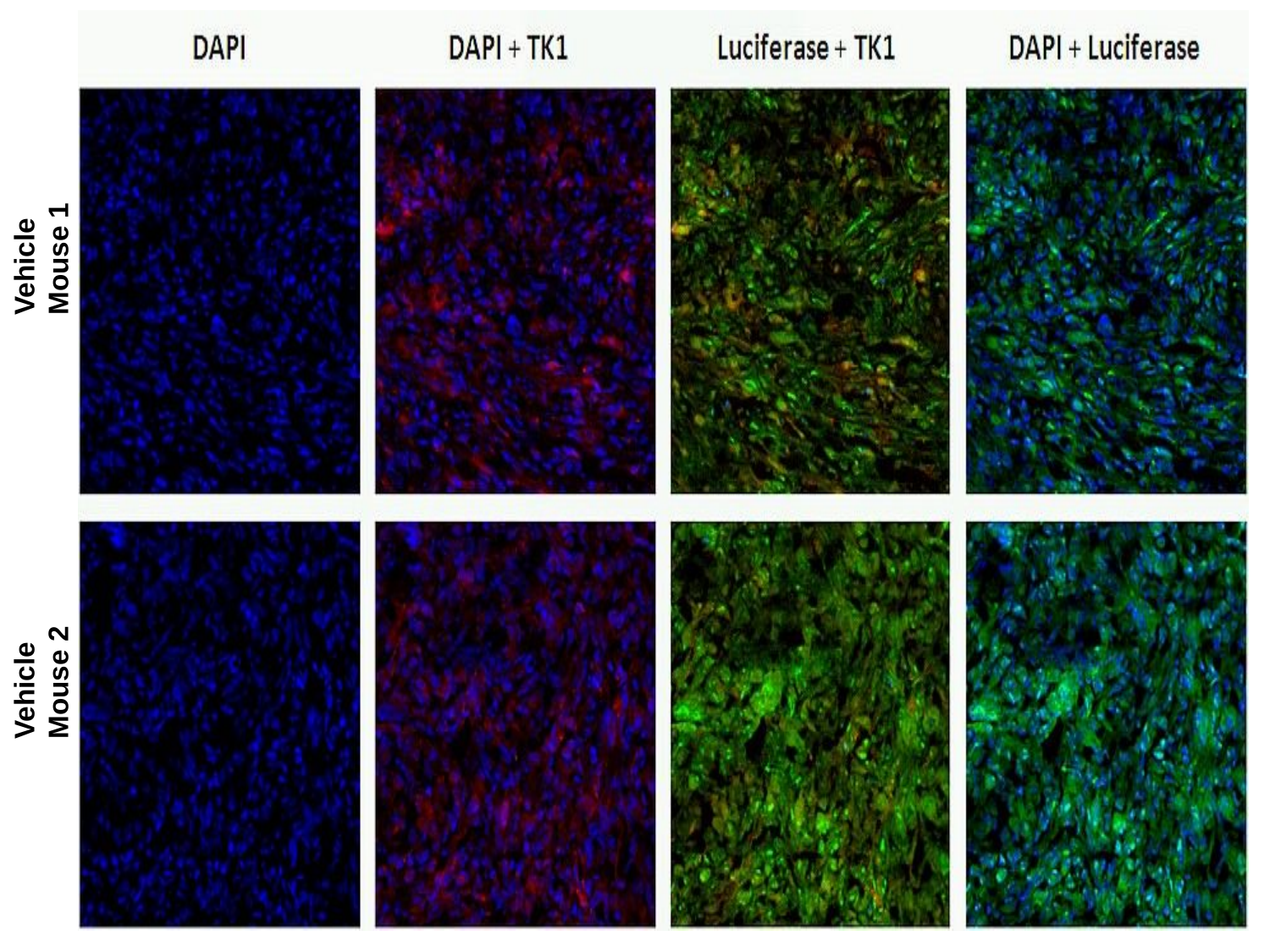

Vehicle
Mouse 1
Vehicle
Mouse 2

Supplement: Additional file 3 — Figure S3. Immunofluorescence images of tumor sections. Images of vehicle-treated samples were generated from 4-μm-thick tumor tissue slices adjacent to the tumor sections used to acquire the nanostructure-initiator mass spectrometry (NIMS) images. Representative images at 20× magnification from vehicle-treated tumors (top panel: NIMS Chip 1 vehicle tumor; bottom panel: NIMS Chip 2 vehicle tumor). Displayed areas were selected to be representative of viable tumor regions based on DAPI staining (blue); in addition TK1 and anti-luciferase immunoreactivity (red and green, respectively) are shown. [file 2049-3002-1-4-S3.pptx]
